# Bilateral Renal Tumour as Indicator for Birt-Hogg-Dubé Syndrome

**DOI:** 10.1155/2014/618675

**Published:** 2014-03-20

**Authors:** P. C. Johannesma, R. J. A. van Moorselaar, S. Horenblas, L. E. van der Kolk, E. Thunnissen, J. H. T. M. van Waesberghe, F. H. Menko, P. E. Postmus

**Affiliations:** ^1^Department of Pulmonary Diseases, VU University Medical Center, P.O. Box 7057, 1007 MB Amsterdam, The Netherlands; ^2^Department of Urology, VU University Medical Center, Amsterdam, The Netherlands; ^3^Urologic Oncology and Department of Urology, The Netherlands Cancer Institute, P.O. Box 90203, 1006 BE Amsterdam, The Netherlands; ^4^Department of Clinical Genetics, The Netherlands Cancer Institute, P.O. Box 90203, 1006 BE Amsterdam, The Netherlands; ^5^Department of Pathology, VU University Medical Center, Amsterdam, The Netherlands; ^6^Department of Radiology, VU University Medical Center, Amsterdam, The Netherlands; ^7^Department of Clinical Genetics, VU University Medical Center, Amsterdam, The Netherlands

## Abstract

Birt-Hogg-Dubé (BHD) syndrome is a cancer disorder caused by a pathogenic *FLCN* mutation characterized by fibrofolliculomas, lung cysts, pneumothorax, benign renal cyst, and renal cell carcinoma (RCC). In this case we describe a patient with bilateral renal tumour and a positive familial history for pneumothorax and renal cancer. Based on this clinical presentation, the patient was suspected for BHD syndrome, which was confirmed after molecular testing. We discuss the importance of recognizing this autosomal dominant cancer disorder when a patient is presented at the urologist with a positive family history of chromophobe renal cell cancer or a positive familial history for renal cell cancer and pneumothorax.

## 1. Background

Birt-Hogg-Dubé syndrome (BHDS) was originally described in 1977 and is nowadays known as a rare autosomal dominant cancer disorder characterized by fibrofolliculomas, lung cysts, pneumothorax, benign renal cyst, and renal cell carcinoma (RCC). The mutated gene for BHD encodes the protein folliculin (*FLCN*) which acts as a tumour suppressor and interacts with mTOR and AMPK signalling pathways [1]. Here we report a case of a patient with bilateral renal cancer and a positive familial history for pneumothorax and renal cancer. Based on the bilateral renal tumour and the positive family history for renal cancer and pneumothorax, Birt-Hogg-Dubé syndrome was suspected.

## 2. Case Report

**Figure 1:**
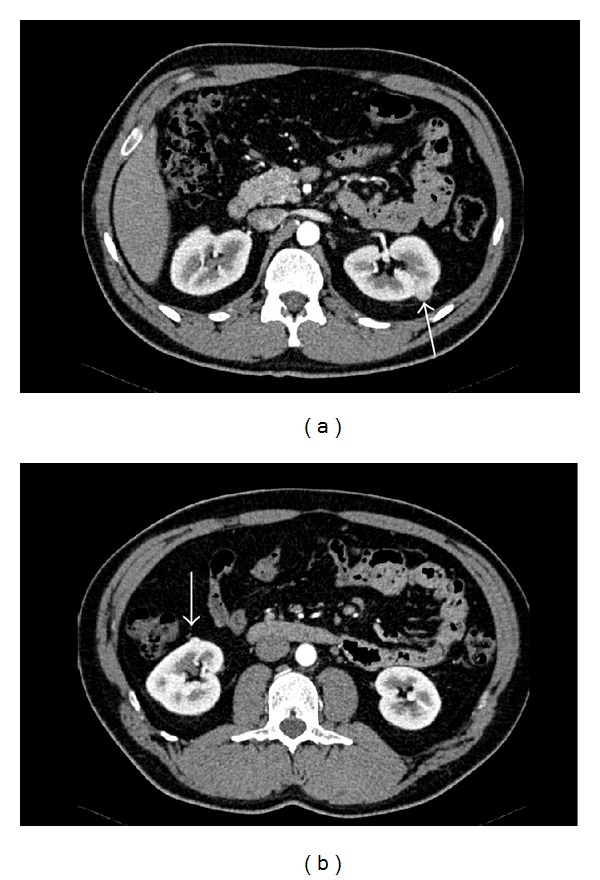
Contrast enhanced CT shows in the arterial phase a hypervascular small lesion in both kidneys, representing two small chromophobe renal cell carcinomas. The tumour in the left kidney (a) was treated by radiofrequency ablation (RFA); for the tumour in the right kidney, (b) follow up was proposed.

**Figure 2:**
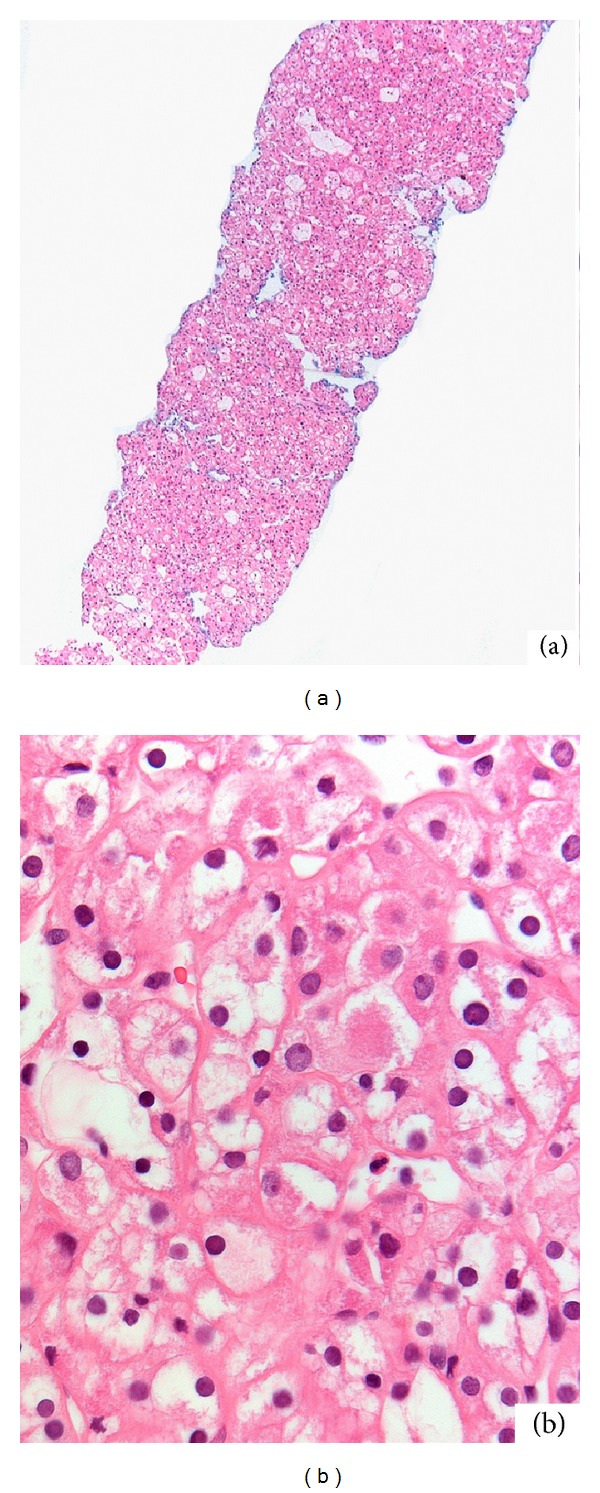
Overview of needle biopsy of tumor in the left kidney ((a): amplification ×2.5) and detail ((b): amplification ×40) of a chromophobe renal carcinoma. Overview of needle biopsy ((a): amplification ×2.5) and detail ((b): amplification ×40) of monotonous cellular pattern with mild nuclear pleomorphy and abundant partly eosinophilic cytoplasm with perinuclear halo, compatible with chromophobe renal cell carcinoma.

In March 2011, a 44-year-old Caucasian male was evaluated for urolithiasis. He had no physical complaints, macroscopic haematuria, or weight loss. His medical and social history were unremarkable; he never smoked. His father had been treated for colorectal cancer; his mother had three episodes of spontaneous pneumothorax and had been treated for a renal tumour. Physical examination of the abdomen showed no abnormalities. Routine laboratory tests were normal. Computed tomography (CT) of the abdomen showed an interpolar tumour in the left kidney, diameter 14 mm (Figure 1(a), arrow), and a second tumour in the upper pole of the right kidney, diameter 8 mm (Figure 1(b), arrow). After a needle biopsy of the largest tumour, revealing a chromophobe renal cell carcinoma (Figures 2(a) and 2(b)), the tumour in the left kidney was treated with radio frequency ablation (RFA). The CT findings in combination with the positive family history for renal cancer and his mother's episodes of pneumothorax suggested Birt-Hogg-Dubé (BHD) syndrome. Sequencing of the* FLCN* gene showed a pathogenic heterozygous frameshift mutation (c.155delc; p.Leu518Phefs∗19)—which has not been described before in literature—and confirmed the diagnosis of Birt-Hogg-Dubé syndrome. The index patient had neither siblings nor children. His parents died years ago; blood or tissue was not available for molecular testing. Frequent follow up by magnetic resonance imaging (MRI) will be performed for evaluation of the small tumour in the right kidney and possible recurrence of the tumour in the left kidney.

## 3. Discussion and Conclusion

Birt-Hogg-Dubé syndrome [OMIM #135150] is a rare autosomal dominant inherited disorder caused by a mutation in the* FLCN* gene located on chromosome 17p11.2, which acts as a tumour suppressor and probably interacts with mTOR and AMPK signalling pathways [1]. BHD is clinically characterized by skin fibrofolliculomas, lung cysts, (recurrent) spontaneous pneumothorax, and renal cancer [2]. In literature, cooccurrence of BHD and a range of tumours, other than renal cancer, has been described, but so far a causal relationship between BHD and these benign and malignant tumours has not been proven [2]. Skin fibrofolliculomas are multiple, dome-shaped, whitish papules located on the scalp, forehead, face, and neck and are found in approximately 90% of families with confirmed BHD syndrome. Dermatologic consultation confirmed multiple fibrofolliculomas on the forehead and face of the index patient. Although cosmetic therapeutic options are limited, case reports suggest that laser ablation, using a YAG or fractional CO_2_ laser, gives temporary improvement [3].

BHD patients have a 50-fold higher risk to develop primary spontaneous pneumothorax (PSP) compared to the normal population. PSP in BHD patients occurs usually after the age of 20, although it has been described already at the age of 7 years. Up to 90% of BHD patients have multiple lung cysts, usually located in the basal regions [4]. In our clinic, we found an estimated penetrance for pneumothorax of 29% (CI: 9–49%) at 70 years of age. BHD patients usually have a normal lung function and no pulmonary symptoms. A CT of the chest performed in our index patient showed no pulmonary cysts.

Renal cell cancer (RCC) is the most lethal of the urologic malignancies, with estimated 273,518 new cases diagnosed and 116,368 patient deaths in 2008 worldwide [5].

RCC can be divided into sporadic and hereditary. The majority is of sporadic origin, presenting normally after the age of 60 as one lesion, while the hereditary type mainly presents as multifocal and bilateral at a far younger age. The most common hereditary renal cancer syndromes are associated with hereditary leiomyomatosis and renal cell carcinoma (HLRCC), Von Hippel-Lindau syndrome (VHL), and hereditary papillary renal carcinoma (HPRC). The prevalence of RCC in (familial) renal cancer is unknown and might be underestimated, since the majority (>80%) of the BHD index patients are referred by a dermatologist.

In a large published series by Pavlovich et al., 34 of 124 individuals (27.4%) with genetic confirmed BHD had a median of 5 renal tumours at a mean age of 50.4 years (range 31–74 years) [6]. In a large series published by Toro et al., 34% of individuals with BHD had renal tumours [4]. The youngest patient with BHD who developed renal cancer was 20 years old [7]. Another study reported a history of renal cancer and metastasis in the same year in a patient with BHD at age 27 [8].

In our Dutch study population published by Houweling et al., we found, among 115 BHD patients, 14 patients with renal cancer and calculated an estimated penetrance for renal cancer of 16% (CI: 6–26%) at 70 years of age [9]. Most lesions are a mixture of solid and cystic components. The largest published histological series of RCC among BHD patients demonstrates that these tumours contain both oncocytoma and chromophobe elements [10]. Houweling and colleagues confirmed these findings, as they found in the majority of RCC tumours cells with granular/floccular eosinophilic cytoplasm, as can be seen in both clear cell carcinoma and chromophobe carcinoma [9].

As the risk of developing RCC is high, imaging and follow up at regular intervals are advised by MRI from the age of 20. The role of ultrasound (US) for detecting renal tumours is still extensively discussed in literature. Surgical treatment is recommended before the largest tumour reaches 3 cm in maximal diameter, which is based on the VHL guideline [8]. Initially, a nephron-sparing surgery should be ideally pursued, which can help prevent chronic renal insufficiency in this patient population. Minimally invasive nephron-sparing techniques such as cryoablation and radio frequency ablation (RFA) are generally accepted as treatment of choice in patients with a unifocal renal lesion. Since BHD patients are at lifelong risk for the development of new tumours, and cryoablation or RFA can complicate both the long term evaluation and surgical management, nephron-sparing surgery is so far the safest and most effective treatment for hereditary renal tumours [11].

In conclusion, in patients with a positive family history of chromophobe renal cell cancer or a positive family history for renal cell cancer and pneumothorax, the diagnosis of BHD should be considered. Therefore, we suggest that easily accessible* FLCN* sequencing should be considered in patients and their families because of the high incidence of renal cancer in BHD patients.

## Abbreviations

FLCN: Folliculin
RCC: Renal cell carcinoma
BHD: Birt-Hogg-Dubé syndrome
CT: Computed tomography
RFA: Radio frequency ablation
mTOR: Mammalian target of rapamycin
AMPK: AMP-activated protein kinase
MRI: Magnetic resonance imaging
US: Ultrasound
PSP: Primary spontaneous pneumothorax
HLRCC: Hereditary leiomyomatosis and renal cell carcinoma
VHL: Von Hippel-Lindau syndrome
HPRC: Hereditary papillary renal carcinoma.
